# Rosiglitazone attenuates the severity of hyperlipidemic severe acute pancreatitis in rats

**DOI:** 10.3892/etm.2013.1255

**Published:** 2013-08-07

**Authors:** BATUR NIYAZ, KAI-LIANG ZHAO, LI-MIN LIU, CHEN CHEN, WEN-HONG DENG, TENG ZUO, QIAO SHI, WEI-XING WANG

**Affiliations:** 1 Department of General Surgery, Renmin Hospital of Wuhan University, Wuhan, Hubei 430060;; 2 Department of General Surgery, Ezhou Central Hospital, Ezhou, Hubei 436000, P.R. China

**Keywords:** hyperlipidemia, acute pancreatitis, peroxisome proliferator activated receptor-γ ligand, cytokine

## Abstract

Peroxisome proliferator-activated receptor-γ (PPAR-γ) ligand regulates adipocyte differentiation and insulin sensitivity, and exerts antihyperlipidemic and anti-inflammatory effects. However, the mechanisms by which PPAR-γ ligands affect hyperlipidemia with severe acute pancreatitis (SAP) have not been fully elucidated. The present study investigated the effects of rosiglitazone, a PPAR-γ ligand, on hyperlipidemia with SAP in a rat model. The hyperlipidemia was induced with a high-fat diet and SAP was induced by the administration of sodium taurocholate (TCA). The hyperlipidemia was shown to aggravate the severity of the sodium taurocholate-induced SAP. However, rosiglitazone demonstrated significant antihyperlipidemic and anti-inflammatory effects in the rats with high-lipid diet-induced hyperlipidemia and SAP.

## Introduction

Hyperlipidemia has been indicated to be associated with acute pancreatitis (AP) in 12–38% of cases worldwide, which has been intensively studied since Speck noted the association between hyperlipidemia and AP in 1865 (1). However, the exact mechanisms by which hyperlipidemia affects the pathogenesis of AP remain uncertain and there are currently no treatment methods for hyperlipidemic severe acute pancreatitis (SAP).

Peroxisome proliferator-activated receptor-γ (PPAR-γ) is one of the most intensively studied nuclear hormone receptors of the last two decades. It is a ligand-activated transcription factor that is expressed in various tissues and cell types, including the pancreas, liver, kidney, adipose tissue and colon (2). Natural ligands of PPAR-γ include fatty acids, arachidonic acid metabolites and prostaglandins. Synthetic ligands include certain nonsteroidal anti-inflammatory agents and a series of antidiabetic agents known as thiazolidinediones (TZDs), including troglitazone, pioglitazone and rosiglitazone (3). PPAR-γ has been shown to be important as a transcriptional mediator in lipid and glucose homeostasis (4–6). A number of studies have demonstrated that PPAR-γ agonists exert potent anti-inflammatory and antioxidant properties. For example, PPAR-γ ligands inhibit proinflammatory cytokine production and macrophage activation, reduce the development of inflammation and tissue injury associated with spinal cord trauma (7), and reduce spinal cord injury, evolution of periodontitis and intestinal ischemia/reperfusion injury (8–10). The present study aimed to evaluate the therapeutic potential of the exogenous PPAR-γ ligand rosiglitazone in rats with hyperlipidemic SAP and to examine its effects on hyperlipidemia associated with a critical illness, in order to explore the mechanism of action.

## Materials and methods

### Reagents

Rosiglitazone was obtained from Cayman Chemical Co. (Ann Arbor, MI, USA) and GW9662 was purchased from Enzo Life Sciences Inc. (Ann Arbor, MI, USA). Additionally, sodium taurocholate (TCA) and dimethyl sulfoxide were purchased from Sigma-Aldrich Co. (St. Louis, MO, USA).

### Animals

Male specific pathogen-free Sprague Dawley rats (weight, 150–200 g) were obtained from the Experimental Animal Center of Hubei Academy of Medical Sciences (Wuhan, China). All animal procedures were approved by the ethics committee of Wuhan University (Wuhan, China) and performed in compliance with the EC regulations and the NIH standards (Guide for the Care and Use of Laboratory Animals, NIH publication, 85–23, revised 1996).

### Animal grouping

**Figure 1.**
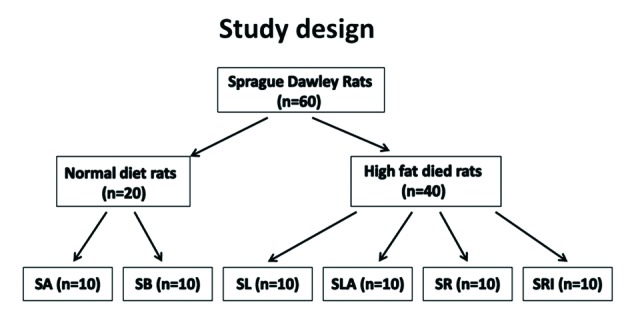
Study design. SA, normal diet + saline; SB, normal diet + sodium taurocholate; SL, hyperlipidemia + saline; SLA, hyperlipidemia + sodium taurocholate; SR, hyperlipidemia + rosiglitazone + sodium taurocholate; SRI, hyperlipidemia + GW9662 + rosiglitazone + sodium taurocholate.

The experimental design is shown in Fig. 1. Sixty animals were randomly divided into two groups, 40 rats received intragastric administration of a high-fat diet (77% normal diet, 20% animal fat and 3% cholesterol) for 2 weeks, which induced experimental hyperlipidemia. The remaining rats received a normal diet and ate freely. Rats receiving the normal diet were divided into 2 groups; the sodium TCA group (SB group, n=10) and the control group (SA group, n=10). The SB group was subjected to sodium TCA-induced SAP and the SA group were injected with saline instead of sodium TCA. Rats with hyperlipidemia were randomly divided into 4 groups: the hyperlipidemia and sodium TCA group (SLA group, n=10); the hyperlipidemia, rosiglitazone and sodium TCA group (SR group, n=10); the hyperlipidemia, GW9662, rosiglitazone and sodium TCA group (SRI group, n=10); and the hyperlipidemia group (SL group, n=10). The SLA and SR groups were subjected to sodium TCA-induced AP and the SR group was additionally treated with 10 mg/kg rosiglitazone by intraperitoneal injection (IP) 1 h prior to sodium TCA. The SRI group were treated in the same way as the SR group with the additional administration of 0.3 mg/kg GW9662 by IP injection 30 min prior to rosiglitazone. The SL group was treated with saline only (Fig. 1).

### Induction of SAP and tissue procurement

Twelve hours prior to the start of the experiment, rats were deprived of food but allowed access to water *ad libitum*. The SAP model was induced by the method by Paszkowski *et al* (11), with improvements. The rats were anesthetized by intraperitoneal injection of 10% chloral hydrate (3 ml/kg). SAP was induced by the retrograde infusion of 5% sodium TCA (1 ml/kg; Sigma-Aldrich Co.) into the ampulla of Vater, transduodenally, using an Angiocath with a standardized pressure-controlled infusion rate under laparotomy. Following infusion, the section of the bile-pancreatic duct entering the duodenum was clipped by a noninvasive Angiocath for 5 min. After checking for bile leakage, the opening in the duodenum lateral wall was sutured. The abdomen was closed, and the rats recovered from the anesthetic and were allowed access to water. All rats were sacrificed by exsanguination 12 h following the induction of pancreatitis, and blood samples were obtained by direct intra-cardiac puncture. The pancreas was removed immediately and the head of the pancreas was fixed in formaldehyde. Tissue sections were paraffin-embedded and continual sections were cut. Portions of this organ were frozen in liquid nitrogen and stored at −80°C until assayed.

### Serum assay

Serum amylase (AMY) activity was measured using an automatic biochemistry analyzer (Olympus Optical Co., Ltd., Tokyo, Japan). Serum triglyceride (TG) and total cholesterol (TC) concentrations were measured in triplicate using commercially available colorimetric assay kits (Diagnosticum Rt, Budapest, Hungary) adapted to 96-well plates, as described previously (12). The accuracy of the assays was monitored using standard lipid controls (Sentinel, Milan, Italy).

### Histopathological examination

Continuous sections of paraffin-embedded pancreatic tissue were stained with hematoxylin and eosin for pathological examination. Morphometric documentation for pancreatic sections using a light microscope (Olympus Optical Co., Ltd., Tokyo, Japan) were evaluated by two independent pathologists, each of whom was blinded to the other’s assessment and recorded their findings on an evaluation form for pancreatic injury. The evaluation of pancreatic injury, including the graded assessment of pancreatic edema, vascular edema, fat necrosis, acinar necrosis and calcification, was determined by the histological score of pancreatic injury (13).

### Determination of intercellular adhesion molecule-1 (ICAM-1) and tumor necrosis factor-α (TNF-α) protein expression by western blot analysis

Frozen pancreatic tissue was mechanically homogenized in 1 ml ice-cold extraction buffer (50 mM Tris-HCl, pH 7.4; 1% NP-40; 0.25% sodium deoxycholate; 150 mM NaCl; 1 mM ethylene diamine tetraacetic acid; 1 mM phenylmethylsulfonyl fluoride; 0.1% sodium dodecylsulfate and 1 *μ*g/ml each of aprotinin and leupeptin). Tissue samples were incubated on ice for 30 min, then centrifuged at 13,000 × g at 4°C for 30 min, and the supernatant was collected and stored at −80°C. The protein concentration of each sample was determined using the Bradford method with bovine serum albumin as the standard. Protein samples (40 *μ*g) were electro-phoresed using sodium dodecyl sulfate-polyacrylamide gels at 100 V for 120 min. The separated proteins were transferred to a nitrocellulose membrane. Moreover, the membrane was blocked with blocking buffer [Tris-buffered saline (TBS) containing 5% non-fat dry milk and 0.1% Tween-20] for 120 min at room temperature, then washed three times for 10 min each in TBS with 0.1% Tween-20 and incubated with the primary antibodies. The primary antibodies used were goat polyclonal anti-rat ICAM-1 (1:600; Santa Cruz Biotechnology Inc., CA, USA) antibody, rabbit polyclonal anti-rat TNF-α antibody (1:1,000; Abcam, MA, USA) and rabbit polyclonal anti-rat β-actin antibody (1:1,000; Cell Signaling Technology Inc., Danvers, MA, USA), and were stored overnight at 4°C. Tissue samples were washed three times for 10 min each with TBS containing 0.05% Tween-20 (TBST) and the membranes were incubated with secondary antibodies, horseradish peroxidase (HRP)-conjugated goat anti-rabbit or rabbit anti-goat immunoglobulin G (1:3,000, Pierce Biotechnology, Rockford, IL, USA) for 1 h at room temperature. Following repeated washings with TBST, the antibody-antigen complex was detected with enhanced chemiluminescence reagent (Immobilon Western HRP Substrate; Millipore Corp., Bedford, MA, USA) and scanned for densitometric analysis using a bio-image analysis system (Bio-Rad Laboratories Inc., Baltimore, MD, USA) for quantification. The results from each experimental group are expressed as the relative integrated intensity of ICAM-1 and TNF-α compared with the β-actin band densities in the same batch.

### Statistical analysis

Data are expressed as the mean ± standard deviation. Statistical analysis was performed with SPSS for Windows, version 17.0 (SPSS, Inc., Chicago, IL, USA). Means of the different groups were compared using one-way analysis of variance. P<0.05 was considered to indicate a statistically significant difference.

## Results

### Serum lipids

Following the 2-week high-fat diet period, the rats with hyperlipidemia were heavier than the rats that had received a normal diet, although not significantly so. However, the 2-week high-fat diet significantly increased the serum cholesterol and TG levels from 1.72±0.13 and 0.61±0.12 mmol/l to 10.86±1.47 and 1.24±0.28 mmol/l, respectively (P<0.05; Table I).

### Histological analysis of pancreatic tissue

**Figure 2.**
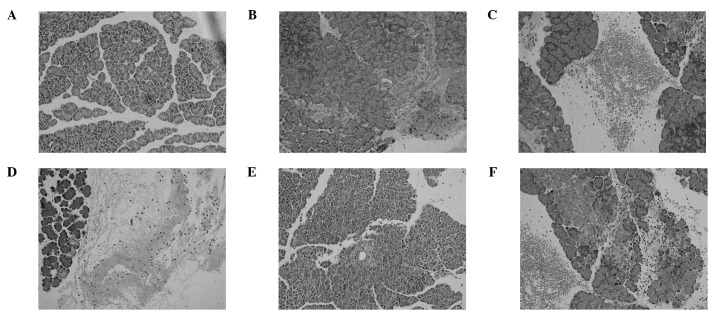
Morphological changes of pancreatitis. Hematoxylin and eosin-stained sections were examined under a light microscope (original magnification, x200) in groups (A) SA, (B) SL, (C) SB, (D) SLA, (E) SR and (F) SRI. SA, normal diet + saline; SB, normal diet + sodium taurocholate; SL, hyperlipidemia + saline; SLA, hyperlipidemia + sodium taurocholate; SR, hyperlipidemia + rosiglitazone + sodium taurocholate; SRI, hyperlipidemia + GW9662 + rosiglitazone + sodium taurocholate.

**Figure 3.**
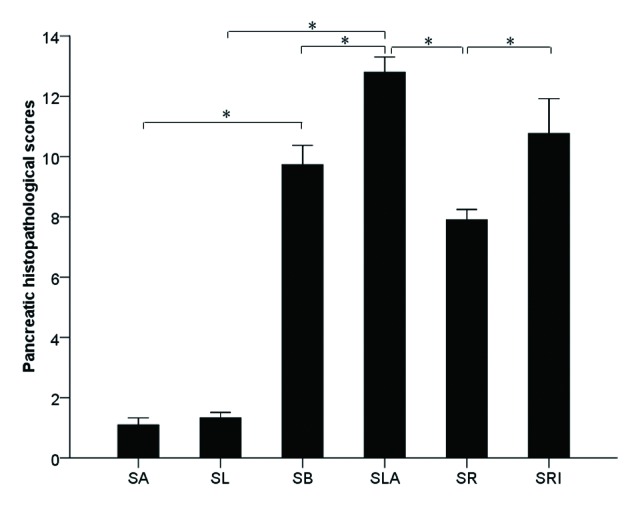
Histopathological scores of pancreatic tissue in all groups. Scores were based on edema, inflammation, hemorrhage and necrosis. One-way analysis of variance was used for statistical analysis. ^*^P<0.05.

Representative histological sections of pancreatic tissue are shown in Fig. 2. The histological severity of pancreatitis was measured with a validated scale (pancreatitis score) based on the degree of edema, inflammatory cell infiltration, hemorrhage and necrosis. No pathological injury in the rats of the SA and SL groups was observed. The total pancreatitis score of the SLA group was significantly higher compared with that of the SB group (P<0.05). Pretreatment with rosiglitazone significantly reduced the inflammatory changes in the SA group compared with those of the SR group (P<0.05); however, the total pathological scores of the SLA and SRI groups were not significantly different (P>0.05; Fig. 3).

### ICAM-1 protein expression

**Figure 4.**
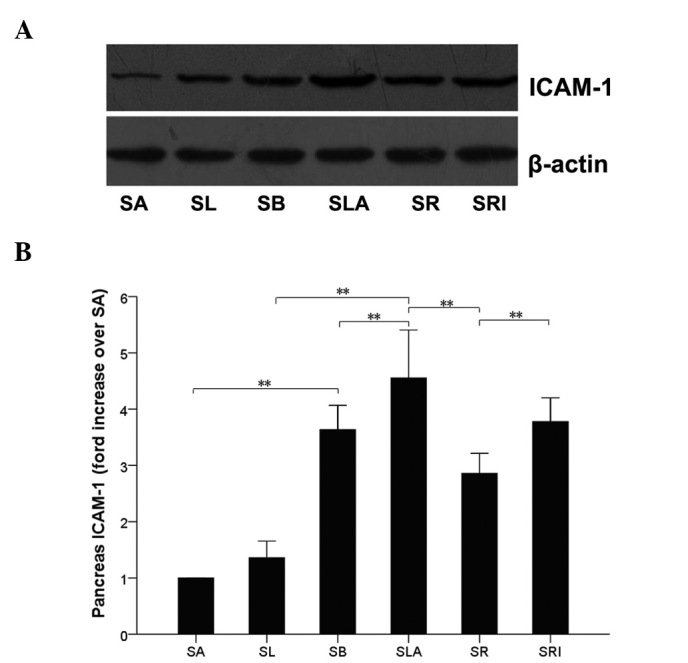
Intercellular adhesion molecule-1 (ICAM-1) expression in the pancreatic tissue in all subgroups detected by western blot analysis. For statistical evaluation, one-way analysis of variance was used. ^**^P<0.05.

In a rat model of SAP similar to that used in the present study (14), the protein expression level of ICAM-1 in the pancreas peaked at 12 h. In the present study, western blot analysis of pancreatic tissues also identified ICAM-1 expression at 12 h following the induction of SAP (Fig. 4). The expression level of ICAM-1 in the pancreatic tissues was significantly higher in the SLA group than in the SL group (P<0.01), and higher in the SB group than in the SA group. The ICAM-1 protein expression level was greatly reduced in the SR group compared with that of the SLA group (P<0.01), and showed no significant difference when compared with those of the SA and SL groups (P>0.05). Furthermore, no significant differences were observed between the SLA and SRI groups with regard to ICAM-1 protein expression (P>0.05). However, the expression of ICAM-1 in the pancreatic tissues was significantly increased in the SLA group compared with that of the SB group (P<0.05; Fig. 4).

### TNF-α protein expression

**Figure 5.**
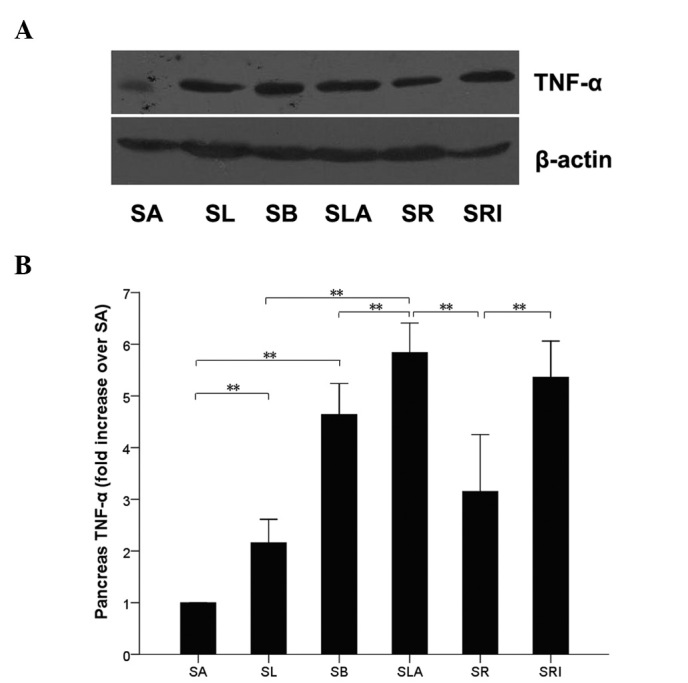
Tumor necrosis factor-α expression in the pancreatic tissue in all subgroups detected by western blot analysis. For statistical evaluation, one-way analysis of variance was used. ^**^P<0.05.

Western blot analysis of the pancreatic tissue (Fig. 5) identified that the protein expression level of TNF-α was significantly higher in the SLA group than in the SL group (P<0.01), and higher in the SB group than in the SA group. The TNF-α protein level was greatly reduced in the SR group compared with that in the SLA group (P<0.01), and showed no difference when compared with those of the SA and SL groups (P>0.05). Furthermore, no significant difference in TNF-α expression between the SLA and SRI groups was observed (P>0.05). However, TNF-α protein expression levels were significantly increased in the SLA group compared with those of the SB group (P<0.05; Fig. 5).

## Discussion

The present study demonstrated that a high-fat diet did not damage the exocrine pancreas; however, it aggravated the severity of sodium TCA-induced SAP. The discrepancies between the results of this study and those from previous studies may be explained by methodological differences (15–17). In isolated *ex vivo* perfused dog pancreas, hyperlipidemia was observed to induce histological and serological alterations of acute pancreatitis (15). Endogenous hyperlipidemia was observed to intensify the course of acute edematous and necrotizing pancreatitis in the rat (16), while exogenous triglycerides increased the pancreatic damage in acute edematous and necrotizing pancreatitis, initiated via different pathogenetic pathways in the isolated perfused pancreas (17).

PPAR-γ is a transcription factor belonging to the nuclear hormone receptor superfamily (18). PPAR-γ activation usually regulates lipid metabolism, glucose homeostasis and influences cell proliferation and differentiation (19–21). In addition, studies have demonstrated that PPAR-γ ligands exhibit anti-inflammatory effects by modulating the production of inflammatory mediators (22,23). Therefore, PPAR-γ ligands may have therapeutic effects on inflammatory diseases, including SAP. The TZDs pioglitazone and rosiglitazone have been approved by the US Food and Drug Administration to control blood glucose levels in patients with type 2 diabetes. TZDs have also been demonstrated to be beneficial in several inflammatory diseases. Rosiglitazone has shown to have protective effects against hyperoxia-induced lung injury (24). Therefore, the anti-inflammatory effects of TZDs may be a general characteristic of PPAR-γ activation, as this phenomenon has been indicated in certain models of critical diseases (25,26). The PPAR-γ natural agonist 15d-prostaglandin J2 was shown to exhibit protective effects in the brain against ischemia-reperfusion injury (27) and to reduce the development of inflammation in mice with acute lung injury induced by lipopolysaccharides (28).

In the present study, the effects of using rosiglitazone as a therapeutic agent to treat rats with hyperlipidemic SAP were evaluated. The results showed that rosiglitazone not only decreased the severity of pancreatic damage but also significantly decreased the levels of serum AMY, TC and TG, and inhibited the release of the proinflammatory cytokines TNF-α and ICAM-1 in pancreatic tissue. This study has demonstrated that rosiglitazone represents a potential therapeutic strategy for the treatment of hyperlipidemic SAP.

In this study, rats of the SR group were intraperitoneally treated with 10 mg/kg rosiglitazone 1 h prior to the induction of SAP, with the aim of preventing inflammation and hyperlipidemic effects. However, the SRI group who were treated with PPAR-γ antagonist GW9662, showed a significant increase in tissue damage compared with that observed in the SL and SR groups. This suggested that PPAR-γ activation by its endogenous ligands is an essential anti-inflammatory event that may be enhanced by providing exogenous agonists. The histological score of pancreatic injury from each group supports the hypothesis that rosiglitazone exerts protective effects in rats with hyperlipidemic SAP.

PPAR-γ activation regulates lipid metabolism and glucose homeostasis, as well as affecting cell proliferation and differentiation (19–21). The definite mechanisms by which PPAR-γ ligands affect hyperlipidemic SAP remain unclear. Previous studies have demonstrated that the effects of PPAR-γ may be attributed to the inhibition of proinflammatory transcription factors, such as activator protein-1, signal transducers and activators of transcription and nuclear factor-κB (NF-κB) (29), modulation of p38 mitogen-activated protein kinase activity and partitioning of the corepressor of B-cell lymphoma 6 (BCL-6) (30). Lim *et al* (31) identified that baicalin-induced PPAR-γ expression inhibited age-related inflammation through blocking proinflammatory NF-κB activation. In a direct interaction model, PPAR-γ is actively exported from the nucleus into the cytosol through interaction with NF-κB, which results in an alteration in the expression of proinflammatory genes, including TNF-α, vascular cell adhesion protein-1, interleukin-1β (IL-1β) and IL-6 (32). Therefore, PPAR-γ ligands may exert potent anti-inflammatory properties by inhibiting NF-κB activation. Moreover, NF-κB is important for the inflammatory response of AP and the intervention against NF-κB activation eliminates the induced overexpression of inflammatory cytokines, TNF-α and ICAM-1.

ICAM-1, an inducible cell transmembrane glycoprotein of the immunoglobulin supergene family, usually expressed at low levels on the surface of endothelial cells, acts as an important component in the inflammatory response for recruitment of leukocytes to sites of inflammation. Several studies have demonstrated that ICAM-1 is upregulated during inflammation, asthma, rheumatoid arthritis and lung injury (33–36), and the expression of ICAM-1 is mediated by various inflammatory cytokines, particularly TNF-α (37). Furthermore, ICAM-1 is known to be upregulated in AP and it recruits neutrophils into the pancreas and distant organs, which inhibits the development of the disease (38,39). In the present study, ICAM-1 protein expression was significantly increased in the pancreatic tissue of rats in the SB and SLA groups. In addition, pretreatment with rosiglitazone markedly attenuated the expression of ICAM-1 in the pancreas tissues of rats in the SR group. Cuzzocrea *et al* also identified that rosiglitazone attenuates the severity of acute inflammation through the reduction of ICAM-1 protein expression in the pancreatic tissue of cerulein-treated mice (40).

A previous study has indicated that, among various inflammatory mediators related to SAP, TNF-α is key in the pathogenesis of SAP (41). It is secreted from severely damaged acinar cells, macrophages and monocytes, provides the regulation of cellular apoptosis and increases the sequestration of pancreatic leukocytes. Various studies have shown that the level of TNF-α, which is a proinflammatory cytokine, is increased in models of AP (42–44). Results from the present study support these previous findings. TNF-α levels in the SB and SLA groups were significantly higher compared with those of the SA and SL groups. Therefore, prevention of TNF-α activity has a beneficial effect on the severity of AP (23,44). In the present study, the prophylactic administration of rosiglitazone markedly reduced TNF-α expression in the pancreas. Moreover, there were no significant differences in the level of TNF-α between the SR and SRI groups, which may indicate a further suppressive effect of rosiglitazone.

In summary, the present study demonstrated that rosiglitazone, a specific PPAR-γ ligand, markedly reduced the severity of pancreatic injury in rats with hyperlipidemic SAP. The results of this study suggest a potential role of rosiglitazone as a therapeutic agent against hyperlipidemic SAP. However, there were several limitations to this study. As rosiglitazone pretreatment is likely to be unsuitable for use in clinical practice, further studies are required to confirm the effects of rosiglitazone treatment on hyperlipidemic SAP.

## Figures and Tables

**Table I.** Serum AMY, TC, TG, HDL and LDL levels.

Group	No. of rats	AMY (U/l)	TC (mmol/l)	TG (mmol/l)	HDL (mmol/l)	LDL (mmol/l)
SA	10	1200.0±0.0	1.72±0.13	0.61±0.12	0.82±0.04	0.66±0.04
SL	10	1139.3±35.6	10.86±1.47^a^	1.24±0.28^a^	1.61±0.11^a^	7.85±1.06^a^
SB	10	5922.2±925.9^a^	2.82±0.24	0.54±0.08	0.97±0.09	1.76±0.14
SLA	10	6501.9±3771.0^b,c^	10.42±0.95^c^	1.36±0.13^c^	1.64±0.07^c^	6.31±0.72^c^
SR	10	2006.9±331.9^b,d^	4.36±0.99^b,d^	0.58±0.12^b,d^	1.29±0.17	8.21±0.50
SRI	10	5892.2±474.3^e^	11.08±1.05^e^	1.58±0.12^e^	1.19±0.07	6.40±0.76

Data are expressed as means ± standard deviation. For statistical evaluation, one-way analysis of variance was used.

a P<0.05, compared with the SA group;

b P<0.05, compared with the SL group;

c P<0.05, compared with the SB group;

d P<0.05, compared with the SLA group;

e P<0.05, compared with the SR group. SA, normal diet + saline; SB, normal diet + sodium taurocholate; SL, hyperlipidemia + saline; SLA, hyperlipidemia + sodium taurocholate; SR, hyperlipidemia + rosiglitazone + sodium taurocholate; SRI, hyperlipidemia + GW9662 + rosiglitazone + sodium taurocholate; AMY, amylase; TC, total cholesterol; TG, triglycerides; HDL, high-density lipoprotein; LDL, low-density lipoprotein.
